# Belhassen anterior fascicular ventricular tachycardia: a case in a black African

**DOI:** 10.1002/ccr3.1535

**Published:** 2018-04-17

**Authors:** Soulemane Pessinaba, Messan Agbetiafa, Messanvi Aloumon, Komlavi Yayehd, Yawo Molba Dodzi Atti, Findibe Damorou

**Affiliations:** ^1^ Cardiology Department Campus Teaching Hospital Lome Togo; ^2^ University of Lome Lome Togo

**Keywords:** African, Belhassen tachycardia, right axis, right bundle branch block, verapamil

## Abstract

Belhassen ventricular tachycardia can be characterized by a complete right branch block and a right axial deviation. This type, although rare, must be recognized to properly treat the patient, as verapamil treatment is effective.

## Introduction

Sustained monomorphic ventricular tachycardia is most often associated in 90% of cases with structural heart disease, including ischemic heart disease, congenital heart disease, valvular dysfunction, and myocardial dysfunction 1, 2. The remaining 10% are referred to as “idiopathic” VT, as they represent VT in the absence of identifiable structural disease 1, 2. The most common type of idiopathic VT (approximately 80%) originates from the outflow tract of either a ventricle or a coronary cusp. The remaining types comprise VTs originating close to the left posterior fascicle or, more rarely, the left anterior fascicle 3 and are called Belhassen ventricular tachycardia. These tachycardias are described in Asians and Westerners 1, 2, 3, 4. We report a rare case of anterior fascicular idiopathic tachycardia of Belhassen in a black African.

## Case Presentation

**Figure 1:**
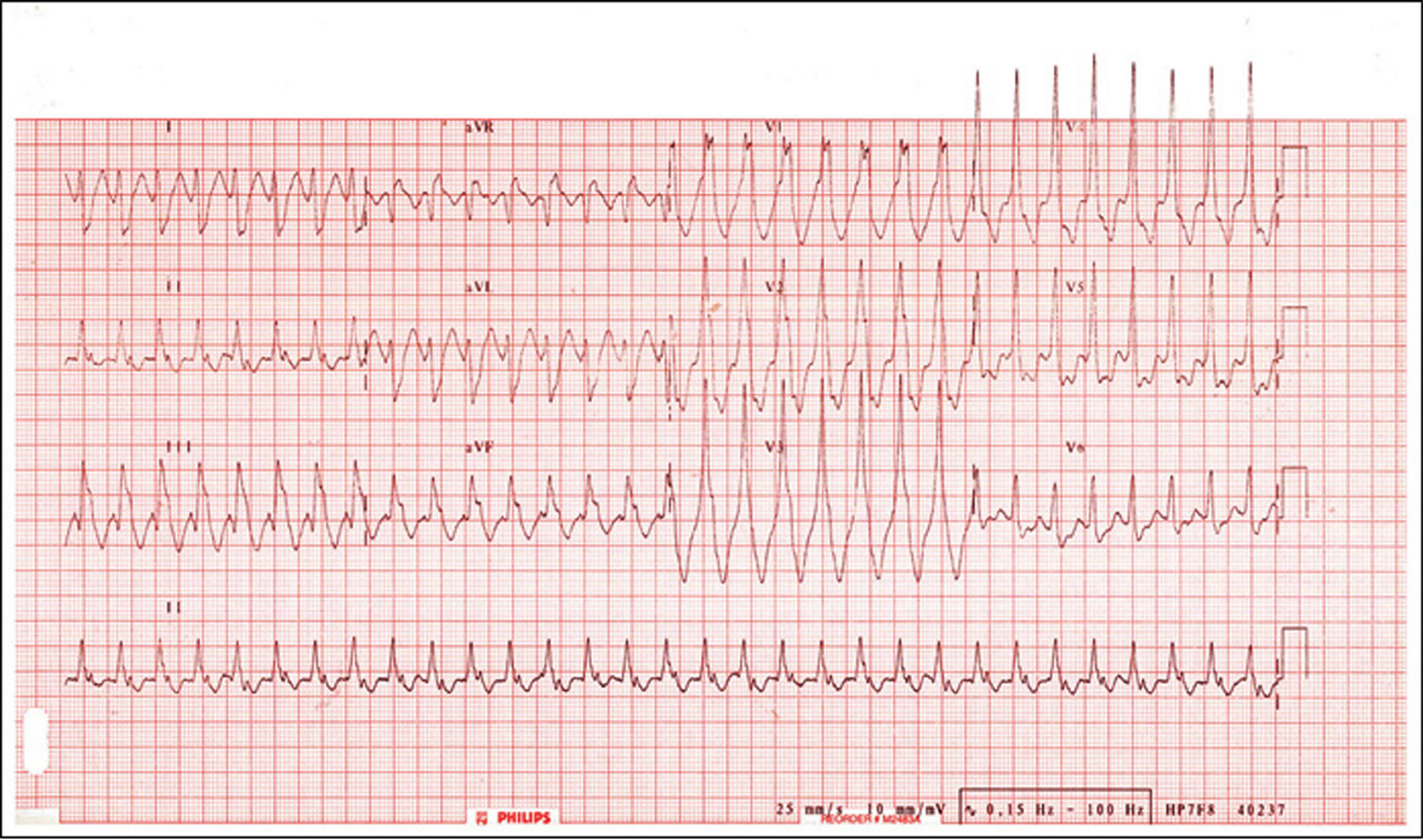
ECG indicating Belhassen tachycardia as evidenced by a rate of 209 bpm and a right bundle branch block (RBBB) and posterior fascicule bundle block.

**Figure 2:**
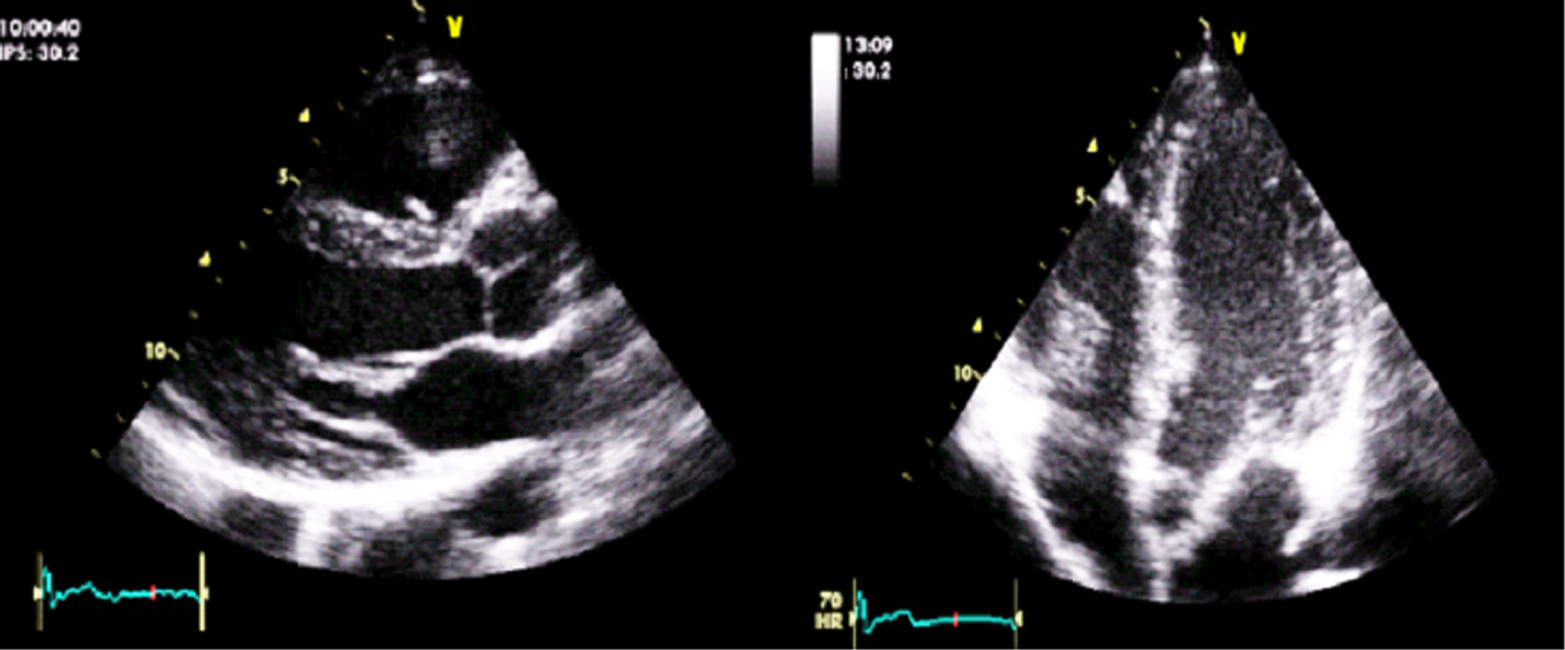
Echocardiogram findings indicated no significant structural heart disease.

**Figure 3:**
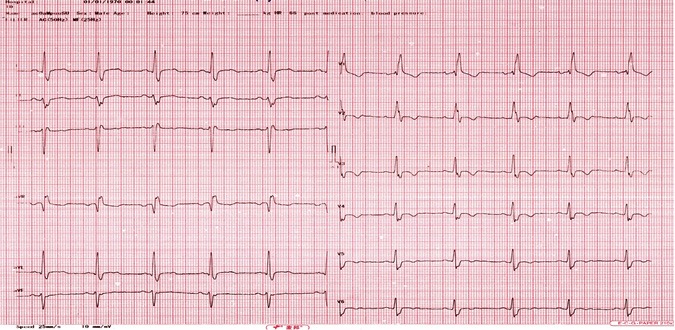
ECG after intravenous verapamil showing sinus rhythm, incomplete right bundle branch block, and Left‐axis deviation (−40°).

Mr. T.P. is a 49‐year‐old patient of Togolese nationality, who has been admitted to our department for throbbing, NYHA Stage II stress dyspnea and intense angular chest pain. He had no personal history of cardiovascular disease. No heart disease or sudden death in the family. The admission examination had found a conscious patient, in good general condition with a blood pressure of 122/77 mmHg, a SaO_2_ at 99% in ambient air, an obesity with BMI at 33 kg/m^2^, a regular tachycardia at 200 bpm without added noise. There were no peripheral signs of heart failure. The emergency electrocardiogram recorded a regular tachycardia at 209 bpm at wide QRS with a complete right bundle branch block and 150° right axis (Fig. 1). There was complete atrioventricular dissociation. In total, the electrocardiogram recorded monomorphic ventricular tachycardia with a complete right bundle branch block aspect and posterior fascicular block. In the emergency, we had carried out an external electrical shock biphasic 150 J which allowed a return in sinus rhythm. Transthoracic echocardiography showed normal sized heart chambers with a left ventricular ejection fraction at 68%. There were no other structural anomalies objectivized (Fig. 2). The diagnosis of anterior fascicular ventricular tachycardia of Belhassen was then discussed. Thirty minutes later, the patient experienced a recurrence of ventricular tachycardia with identical QRS as those on the 1st ECG. The patient was this time put on verapamil injection, which allowed to obtain a sinus rhythm at 66 bpm, an incomplete right block and an axis at −40° (Fig. 3). The biological assessment performed including glycaemia, creatinine, blood ionogram, troponin I, calcium, magnesium, and blood count was normal. The 24‐h ECG Holter performed the next day had noted right ventricular extrasystole with QRS identical to ventricular tachycardia.

The patient was placed on oral verapamil, followed by bisoprolol 1 month later. The evolution was marked by two recurrences. We therefore combined verapamil with bisoprolol, which allowed us to maintain a permanent sinus rhythm with a current decline of 15 months.

## Discussion

Belhassen tachycardia is a rare left ventricular arrhythmia that was described as a unique electrophysiological entity in 1981 by Belhassen et al. 5.Commonly referred to as fascicular or intrafascicular tachycardia, it is generally characterized by a right bundle branch block pattern and left‐axis deviation 3 reflecting its origin at the left posterior fascicle. Exceptionally, it originates at the left anterior fascicle. This is the case in our patient in whom the ECG showed an aspect of right bundle branch block pattern and a straight axis at 150° evoking a posterior fascicular bundle branch block. It is a tachycardia described in the West and much more in Asia. To our knowledge, no case has yet been described in a black African.

Patients are typically young and healthy men; their first episode often occurs in adolescence 5. Symptoms usually present are palpation, dizziness, fatigue, and shortness of breath, and patients are generally hemodynamically stable 1, 2. Our patient had palpitations, dyspnea, and chest pain and was hemodynamically stable.

The mechanism of VT is assumed to be macro‐reentry within the Purkinje network in most patients 6, 7, although in rare cases triggered activity could be operative 8. It is believed that this reentry tachycardia stems from abnormal Purkinje fibers because of its dependence on the slow conduction of calcium in partial depolarization 9. This is significant because Purkinje fibers are targeted and guide therapy in acute management of Belhassen tachycardia through the use of verapamil. It is known that idiopathic ventricular tachycardias depend on slow entry calcium in partially depolarized Purkinje fibers, thus making verapamil the ideal first‐line treatment 10.

Typical pharmacological treatment is verapamil. Intravenous verapamil has been effective in treating the ventricular tachycardia, and oral verapamil has had variable results in preventing recurrences 11. Beta‐blocker has been effective both in treating Belhassen ventricular tachycardia and in preventing recurrences 4, 12. Patients with recurrent refractory episodes of VT may be referred for radiofrequency ablation 1, 2. Our patient presented two TV recurrences with bisoprolol. In the absence of interventional treatment in our country, we have combined bisoprolol and verapamil oral which allowed to maintain a sinus rhythm with 15 months decline.

## Conclusion

Belhassen ventricular tachycardia, also known as idiopathic ventricular tachycardia, is a rare condition. The feature of our case lies in the fact that it is the exceptional type of anterior fascicular ventricular tachycardia and is the first case described to our knowledge in a black African.

## Conflict of Interest

No conflict of interest.

## Authorship

SP: wrote and drafted the manuscript, discussed the importance of ECG initiated this study, and was the operator who made the transthoracic echocardiography. MAg and MAl: made the review of the literature and wrote the article. KY: exams and interpretation. YMDA: did the review of the literature, corrected this article, and made contributions. FD: did the final corrections before the article be submitted. All authors read and approved the final manuscript.
